# Suicide amongst psychiatric in-patients who abscond from the ward: a national clinical survey

**DOI:** 10.1186/1471-244X-10-14

**Published:** 2010-02-03

**Authors:** Isabelle M Hunt, Kirsten Windfuhr, Nicola Swinson, Jenny Shaw, Louis Appleby, Nav Kapur, the National Confidential Inquiry into Suicide and Homicide by People with Mental Illness

**Affiliations:** 1 National Confidential Inquiry into Suicide and Homicide by People with Mental Illness, Centre for Suicide Prevention, Jean McFarlane Building, University of Manchester, Manchester, M13 9PL, UK

## Abstract

**Background:**

Suicide prevention by mental health services requires an awareness of the antecedents of suicide amongst high risk groups such as psychiatric in-patients. The goal of this study was to describe the social and clinical characteristics of people who had absconded from an in-patient psychiatric ward prior to suicide, including aspects of the clinical care they received.

**Methods:**

We carried out a national clinical survey based on a 10-year (1997-2006) sample of people in England and Wales who had died by suicide. Detailed data were collected on those who had been in contact with mental health services in the year before death.

**Results:**

There were 1,851 cases of suicide by current psychiatric in-patients, 14% of all patient suicides. 1,292 (70%) occurred off the ward. Four hundred and sixty-nine of these patients died after absconding from the ward, representing 25% of all in-patient suicides and 38% of those that occurred off the ward. Absconding suicides were characterised by being young, unemployed and homeless compared to those who were off the ward with staff agreement. Schizophrenia was the most common diagnosis, and rates of previous violence and substance misuse were high. Absconders were proportionally more likely than in-patients on agreed leave to have been legally detained for treatment, non-compliant with medication, and to have died in the first week of admission. Whilst absconding patients were significantly more likely to have been under a high level of observation, clinicians reported more problems in observation due to either the ward design or other patients on the ward.

**Conclusion:**

Measures that may prevent absconding and subsequent suicide amongst in-patients might include tighter control of ward exits, and more intensive observation of patients, particularly in the early days of admission. Improving the ward environment to provide a supportive and less intimidating experience may contribute to reduced risk.

## Background

Absconding, or going absent without leave, is a common feature within psychiatric wards, with rates of between 34% and 39% cited [1,2]. Some of the adverse consequences of absconding include loss of treatment, violence to others, self-neglect, self-harm, and suicide [3-5]. Controlled studies have found absconding to be a significant risk factor for suicide amongst psychiatric in-patients [6-8]. In the UK, Powell and colleagues [9] reported that 63% of in-patients who died by suicide outside of the hospital site were absent without authorised leave at the time of death. Other studies have reported lower (36% to 40%), but still substantial, rates of absconding when the suicide occurred [6,8,10].

There have been no detailed studies describing the characteristics of patients who have absconded from psychiatric in-patient wards and subsequently died by suicide. Our aims were: firstly, to describe a national, consecutive series of suicide cases by people under mental health care who had absconded from the ward; secondly, to compare the social and clinical features of these suicide cases with those who were on leave or who had left the ward with staff agreement. The study was carried out as part of the National Confidential Inquiry into Suicide and Homicide by People with Mental Illness [11].

## Methods

The methods used in the National Confidential Inquiry have been described in detail elsewhere [12,13]. Briefly, data collection involved three stages. First, the collection of a comprehensive national sample of deaths in England and Wales receiving a verdict of suicide or open verdict from the Office for National Statistics (ONS). Second, information on whether the deceased within the sample had been in contact with health services in the 12 months before death was obtained from the hospitals and community trusts providing mental health services in the deceased's district of residence. Third, clinical data about people who had been in contact with services ('Inquiry cases') were obtained by sending a questionnaire to the responsible consultant psychiatrist. The questionnaire consisted of sections covering social/demographic characteristics, clinical history, details of the suicide, aspects of care, details of final contact with services, and the respondents' views on prevention. The social and clinical items reflected many of the most frequently reported risk factors for suicide. The majority of the items were factual, while a number (e.g., compliance with medication) were based on the judgements of clinicians. Ethical approval was obtained from the South Manchester Medical Research Ethics Committee.

The cases presented here consist of suicides and self-poisoning/self-injury open verdicts registered by ONS from January 1, 1997 until December 31, 2006. Open verdicts were included as most are thought to be suicide cases and are conventionally used in suicide rate estimation in the UK [14,15].

### Statistical analysis

The main findings are presented as proportions with 95% confidence intervals (CIs). Subgroup analysis involved the use of Chi-square tests (unless any cell had an expected frequency less than 5 in which case a Fisher exact test was used). A 2-sided *p *value of < 0.05 was considered as statistically significant. If an item of information was not known for a case, the case was removed from the analysis of that item; the denominator in all estimates is therefore the number of valid cases for each item. Analysis was carried out using Stata 10.0 software [16].

## Results

Over the study period from January 1, 1997 until December 31, 2006, we received notifications of 50,352 cases of suicide, including 34,891 cases in which the coroner's verdict was suicide and 15,461 open verdicts or deaths from undetermined cause. Of these, 13,331 (26%) were confirmed as having been in contact with mental health services in the year prior to death. Completed questionnaires were received on 13,066 cases, a response rate of 98%.

There were 1,851 cases who were current in-patients at the time of suicide, representing 14% of all suicide cases (an average of 185 deaths per year). The number and proportion of in-patient suicides has significantly declined over the 10-year study period, from 221 (17%) cases in 1997 to 144 (12%) in 2006 (likelihood ratio χ^2 ^test for linear trend 16.3 (1 df), p < 0.001). Thirty percent (546 cases) of in-patient suicides took place on the ward itself; 1,292 cases (70%) occurred away from the ward, and in 13 cases (0.7%) the place of death was unknown. Of those who died away from the ward, 469 (38%) had absconded, and 761 (62%) were either on authorised leave or off the ward with staff agreement when the suicide occurred (referred to as 'agreed leave' cases). Over the study period, whilst the number of suicides after absconding had fallen, the proportion showed no clear pattern, fluctuating from 40% (52 cases) in 1997, to 31% (40 cases) in 2003, and 38% in 2006 (35 cases). On average, these patient suicides occurred 50 times per year.

### Type of ward

The majority of absconders were on a general psychiatry open ward (393 cases, 86%); 27 (6%) a rehabilitation unit; 11 (2%) a psychiatric intensive care ward, 5 (1%) a secure unit, and 21 (5%) were on 'other' specified wards (for example, women only crisis unit). In 12 (3%) cases, the type of ward was unknown.

### Method of Suicide

Data on the cause of death are summarised in Table 1. Hanging and jumping from a height or in front of a moving vehicle were the main methods used for the sample as a whole. However, those who had absconded were proportionally less likely to die by hanging and self-poisoning compared to those who were on agreed leave, but more often died by jumping and drowning.

**Table 1:** Method of suicide by leave status

	Absconders (*n *= 469)		Agreed leave (*n *= 761)		
Method	N	%	N	%	P-value
Hanging	106	23%	253	33%	< 0.001
Self-poisoning	31	7%	101	13%	< 0.001
Carbon monoxide poisoning	8	2%	27	4%	0.06
Drowning	69	15%	72	9%	0.01
Jumping	228	49%	231	30%	< 0.001
Other *	26	6%	75	10%	0.01

* includes burning, cutting, firearms, electrocution, suffocation and other specified

### Social and behavioural characteristics

Cases of suicide who had absconded were significantly younger than those who were on agreed leave (median age 39, range 17-78 vs. 46, range 15-95; *p *< 0.001). There was no difference between the two groups in terms of gender, ethnicity or living circumstances (Table 2). However, those who had absconded were more likely to be unemployed, unmarried and homeless. Whilst three quarters of both in-patient groups had self-harmed, those who had absconded were more likely to have had a history of violence, alcohol misuse and drug misuse.

**Table 2:** Socio-demographic and behavioural characteristics of in-patient suicide cases by leave status

	Absconders (*n *= 469)		Agreed leave (*n *= 761)		
Feature	N	%	N	%	P-value
***Socio-demographic***					
Male gender	311	66%	488	64%	0.44
Unemployed	222	48%	281	37%	< 0.001
Ethnic minority	29	6%	59	8%	0.29
Unmarried	353	75%	530	70%	0.03
Living alone	193	42%	341	45%	0.24
Homeless	32	7%	28	4%	0.01
***Behavioural***					
History of self-harm	351	76%	562	75%	0.70
History of violence	131	28%	152	20%	0.001
History of alcohol misuse	171	37%	230	31%	0.03
History of drug misuse	167	36%	176	23%	< 0.001

### Clinical characteristics

The diagnostic profile differed between absconders and those on agreed leave (Table 3). Forty percent of those who had absconded were suffering from schizophrenia, significantly more than other cases (26%). They were also more likely to have alcohol dependence but had lower rates of affective disorder. A co-morbid psychiatric condition was common, occurring in approximately half of both groups. A similar proportion of both groups had also been ill for longer than five years, and had multiple previous admissions to psychiatric in-patient care. There was no difference between absconders and those on agreed leave in terms of the number under enhanced levels of aftercare (the Care Programme Approach (CPA); a mechanism which provides supervision by a care co-ordinator and regular multi-disciplinary case reviews to patients with complex health and social care needs). However, non-compliance with medication was a particular feature of patients who had absconded compared to those on agreed leave.

**Table 3:** Clinical characteristics of in-patient suicide cases by leave status

	Absconders (*n *= 469)		Agreed leave (*n *= 761)		
Feature	N	%	N	%	P-value
*Primary diagnosis:*					
Schizophrenia	188	40%	201	26%	< 0.001
Affective disorder	201	43%	451	59%	< 0.001
Alcohol dependence	13	3%	9	1%	0.04
Drug dependence	3	0.6%	2	0.3%	0.31
Personality disorder	30	6%	40	5%	0.39
Any secondary diagnosis	237	51%	355	47%	0.16
Any adverse life event	179	40%	335	45%	0.06
Over 5 previous admissions	124	26%	213	28%	0.55
Duration of history (>5 years)	262	57%	403	54%	0.29
Under enhanced CPA*	330	73%	520	70%	0.34
Non-compliance in last month	114	25%	100	13%	< 0.001

* Care Programme Approach

### Contact with services

Those who had absconded were more likely than those on agreed leave to have been under a medium (checked every 5 to 25 minutes) or high (one-to-one) level of observation (Table 4). However, clinicians had reported significantly more problems in observation of those who had absconded, through either ward design or other patients. Absconders were also more likely, at this final admission, to have been detained under the Mental Health Act (MHA; the legislation by which patients can be confined in hospital for assessment and treatment against their wishes), and to have died within a week of being admitted. Fewer absconders had died during the period when discharge from hospital was being planned.

**Table 4:** Contact with services and risk characteristics of in-patient suicide cases by leave status

	Absconders (*n *= 469)		Agreed leave (*n *= 761)		
Feature	N	%	N	%	P-value
***Contact with services***					
Observation level: high or medium	117	31%	22	9%	< 0.001
Suicide during period of planning discharge	107	28%	358	58%	< 0.001
Died within first week of admission	55	19%	37	8%	< 0.001
Died within local in-patient unit	185	69%	271	64%	0.20
Detained under MHA*	136	29%	130	17%	< 0.001
Observation problems with ward design	93	22%	77	11%	< 0.001
Observation problems with other patients	31	7%	26	4%	0.01
***Risk***					
Symptoms at last contact	311	68%	388	52%	< 0.001
Immediate risk: medium or high	111	25%	79	11%	< 0.001
Long-term risk: medium or high	208	57%	282	48%	0.003
Suicide thought to be preventable	146	33%	149	21%	< 0.001

* Mental Health Act

Proportionally more patients who had absconded had reported abnormalities of mental state at the last contact with the mental health team. These symptoms were most often emotional distress (166 cases, 36% vs. 138 cases, 18%; *p *< 0.001), hopelessness (94 cases, 21% vs. 76 cases, 10%; *p *< 0.001), delusions or hallucinations (87 cases, 19% vs. 59 cases, 8%; *p *< 0.001) and suicidal ideation (71 cases, 16% vs. 54 cases, 7%; *p *< 0.001). Estimates of both short- and long-term risk of suicide were more often considered as moderate or high in patients who had absconded. Clinicians were also more likely to view absconding cases as preventable. The most common suggested factors that could have made the suicide less likely were closer patient supervision (219 cases, 49%), better treatment compliance (118 cases, 26%), increased staff numbers (114 cases, 26%), improved staff communication (97 cases, 21%) and better staff training (93 cases, 21%).

## Discussion

Our findings have confirmed previous studies that a substantial proportion of in-patient suicides occur after absconding from the ward. Over the 10-year study period, whilst the number of in-patient suicides had declined, the proportion who had absconded remained essentially unchanged (an average of 40% of in-patient suicides that occurred off the ward per year). We have shown that these patient suicides had different characteristics to those who died off the ward with staff agreement, particularly in their clinical features. Absconders were characterised by being young, unemployed and homeless. They had high rates of schizophrenia, previous violence and substance misuse. Methods of suicide were more 'violent' compared to other in-patients, with nearly half of absconders dying by jumping. Detention under mental health legislation was more common amongst absconders, as was medication non-compliance. Around a fifth died within the first week of admission. Many had declared their risk through emotional distress, hopelessness, and suicidal ideation. Levels of observation were higher than those on agreed leave, but the ward design and disturbance by other in-patients were more likely to have hindered observation by staff. Clinicians more often viewed absconding cases to be at high suicide risk and to be preventable.

Our results are in keeping with previous studies that have provided a profile of absconders in general, including being younger [3,4], with high rates of schizophrenia [17-19], substance misuse [20], and medication non-compliance [19]. The finding that patients who had been detained under the MHA were more likely to abscond has previously been reported [4,17,18], although this may be a reflection of the higher proportion of in-patients with severe mental illness.

### Methodological issues

The sample size in this study is larger than has been possible in previous clinical studies and data collection is almost complete. Although it is a national study, the Inquiry has several methodological limitations and these have been described elsewhere [13]. Briefly, they include the lack of a comparison sample to draw aetiological conclusions; obtaining information from case records and clinical judgements, rather than standardised methods; and the potential for bias from clinicians' awareness of patient outcome (particularly on variables such as estimation of risk). Further, we could not establish if patients had previously absconded from the ward, an apparent risk factor for future absconding [19,21], nor could we ascertain the length of time between leaving the ward and suicide unless the death occurred within a week of admission.

### Clinical Implications

In-patients may be admitted for management of suicide risk, therefore any in-patient suicides that occur could reflect service quality. The National Patient Safety Agency (NPSA) has described in-patient suicides by hanging from non-collapsible rails as a 'Never Event', i.e. an incident that should not occur if available preventative measures have been implemented [22]. Other measures to prevent in-patient suicide might include regular risk assessments during recovery and prior to granting leave, adequate monitoring of patients, staff training programmes in the management of risk, and improved staff communication [8,23,24]. However, suicide after absconding is problematic, and it is clearly a challenge to prevent patients leaving a general psychiatry open ward. The current findings can, however, inform staff of the clinical characteristics associated with absconding suicides, such as schizophrenia, substance misuse and non-compliance.

How might clinical efforts be concentrated to reduce absconding from in-patient units? Firstly, particular attention could be paid by staff in observing not only the patients themselves but also the ward exits. This could have implications for staffing levels, but improved ward security through video monitoring or swipe card systems to regulate patients' entry and exit, may be effective. Environmental factors are likely to play a part in the level of absconding from wards, therefore we recommend regular reviews of wards for any obstructions to observation, and assess the suitability of these wards for high-risk patients. For those viewed as particularly high risk, staff may wish to consider transfer to a locked Psychiatric Intensive Care Unit (PICU) to ensure a more secure environment.

Secondly, improved observation methods. There is scant evidence regarding the protective effect of close observation [25] and we have shown that a high level of observation may be ineffective in patients who are determined to leave the ward. However, it may be that observation protocols need to be reviewed and specific levels of observation tailored to individual patients during assessment of risk. The first few days post admission are known to be a time of particular suicide risk [8,26], and our finding that absconders were more likely to die in the first week of admission emphasises the need for optimum observation protocols at this early stage. Services could also give greater priority to policies in the event of an absconsion, such as early plans to search the ward and its surroundings, as well as contacting family members, who are known to play a crucial role in encouraging patients to return to hospital [27].

Thirdly, at admission there could be increased focus on engagement and support by staff, with attempts to make the ward environment less oppressive and as non-stigmatising as possible, and encouragement by staff to seek support in times of crises. Indeed, the Institute for Innovation and Improvement [28] recommends services place greater emphasis on creating a ward environment which engages the patient, promotes support and includes a variety of structured and interesting activities. A recent report by the National Mental Health Unit [29] suggests the recording of an absconsion as a clinical incident. Such post-incident reviews may then provide further knowledge of factors that lead to absconding, such as the ward design, ward disturbances, or situational factors that have influenced a patient.

## Conclusion

To conclude, it is clearly challenging to achieve a balance between patient safety and patient autonomy, but the need to protect individuals from harm during a time when they are supposedly in a safe environment should be a principal objective of mental health services.

## Abbreviations

CPA: Care Programme Approach; MHA: Mental Health Act; ONS: Office for National Statistics; NPSA: National Patient Safety Agency; PICU: Psychiatric Intensive Care Unit.

## Competing interests

LA is the National Director of Mental Health for England.

## Authors' contributions

LA conceived of the study. IMH and KW took part in the data collection, supervised by JS, LA and NK. IMH drafted the manuscript and performed the statistical analysis; KW, NS, JS, LA and NK helped to interpret the data and draft the manuscript. All authors read and approved the final manuscript.

## Pre-publication history

The pre-publication history for this paper can be accessed here:

http://www.biomedcentral.com/1471-244X/10/14/prepub
